# A bibliometric analysis of research trends of artificial intelligence in the treatment of autistic spectrum disorders

**DOI:** 10.3389/fpsyt.2022.967074

**Published:** 2022-08-29

**Authors:** Shouyao Zhang, Shuang Wang, Ruilu Liu, Hang Dong, Xinghe Zhang, Xiantao Tai

**Affiliations:** School of Second Clinical Medicine/The Second Affiliated Hospital, Yunnan University of Chinese Medicine, Kunming, China

**Keywords:** autism, artificial intelligence, bibliometrics, knowledge mapping, autistic spectrum disorders

## Abstract

**Objective:**

Autism Spectrum Disorder (ASD) is a serious neurodevelopmental disorder that has become the leading cause of disability in children. Artificial intelligence (AI) is a potential solution to this issue. This study objectively analyzes the global research situation of AI in the treatment of ASD from 1995 to 2022, aiming to explore the global research status and frontier trends in this field.

**Methods:**

Web of Science (WoS) and PubMed databese were searched for Literature related to AI on ASD from 1995 to April 2022. CiteSpace, VOSviewer, Pajek and Scimago Graphica were used to analyze the collaboration between countries/institutions/authors, clusters and bursts of keywords, as well as analyses on references.

**Results:**

A total of 448 literature were included, the total number of literature has shown an increasing trend. The most productive country and institution were the USA, and Vanderbilt University. The authors with the greatest contributions were Warren, Zachary, Sakar, Nilanjan and Swanson, Amy. the most prolific and cited journal is *Journal of Autism and Developmental Disorders*, the highest cited and co-cited articles were Dautenhahn (Socially intelligent robots: dimensions of human-robot interaction 2007) and Scassellati B (Robots for Use in Autism Research 2012). “Artificial Intelligence”, “Brain Computer Interface” and “Humanoid Robot” were the hotspots and frontier trends of AI on ASD.

**Conclusion:**

The application of AI in the treatment of ASD has attracted the attention of researchers all over the world. The education, social function and joint attention of children with ASD are the most concerned issues for global researchers. Robots shows gratifying advantages in these issues and have become the most commonly used technology. Wearable devices and brain-computer interface (BCI) were emerging AI technologies in recent years, which is the direction of further exploration. Restoring social function in individuals with ASD is the ultimate aim and driving force of research in the future.

## Introduction

Autism spectrum disorder (ASD) is a kind of neurodevelopmental disorder characterized by social communication disorder, stereotyped repetitive behavior and narrow interest (1). The pathogenesis of ASD remains unclear, but current studies suggests that it may be associated with genetic inheritance (2), abnormal brain structure (3), environmental factors (4), etc. According to the latest international data, the incidence of ASD has increased from 150:1 in 2000 to 36:1 in 2017 (1), making it the leading cause of disability in children. Recovery from this disease is difficult and expensive. The lifetime cost of supporting an individual with an intellectual disability is $2.4 million in the United States and $2.2 million in the United Kingdom (5), placing a huge burden on families and society.

In recent years, artificial intelligence (AI) has shown impressive development in the treatment of ASD. He humanoid robot represented by Kaspar has significantly enhanced the living ability of children with ASD by simulating human behavior (6). The learning content and learning tendency of ASD children participating in robot intervention courses increased significantly, indicating that AI robots have potential application prospects as classroom assisted learning tools (7). By wearing augmented reality smart glasses, irritability, lethargy, rigid behavior and speech of children with autism were improved (8). Recent studies have also shown that AI technology can not only improve the cognitive and social skills of autistic children, but also have broad application prospects for the rehabilitation of children and adolescents with severe intellectual disability (9).

Bibliometrics is widely used to analyze published scientific literature. In recent years, bibliometric analysis has been carried out on the overall application status and research trend of AI in cardio-cerebrovascular diseases (10), spinal diseases (11) and plastic surgery (12). AI has shown its application value in more and more fields. However, as far as we know, there is no bibliometric analysis on the application of AI in ASD. Therefore, the study aims to analyze the global research situation of ASD treated with AI technology from 1995 to April 2022 through the method of bibliometrics and with the help of literature visualization tools, and present the results in the form of visual maps to comprehensively analyze the research hotspots, future trends, challenges and prospects of the application of AI technology in ASD.

## Methods

All data were obtained from Web of Science (WoS) and PubMed on April 14, 2022. The topics of data retrieval are “Artificial Intelligence” and “Autism Spectrum Disorder” (Table 1). The publication date of the literature is from January 1, 1995 to April 31, 2022. Literature retrieval is not limited to categories, languages, or document types. The search results were analyzed using CiteSpace, VOSviewer, Pajek and Scimago Graphica.

**Table 1:** Search queries.

**Set**	**Result**	**Search Query**
#1	85,942	(TS=((autistic) OR (ASD) OR (pervasive developmental disorders)))
		Indexes=Web of Science, PubMed Timespan=1995-2022
#2	899,849	(TS= ((BCI) OR (brain chip) OR (brain computer interface) OR (brain machine interface) OR (AI) OR (artificial intelligence) OR (robotic)))
		Indexes=Web of Science, PubMed Timespan=1995-2022
#3	1298	#1 AND #2

CiteSpace (version5.6.r4) is a scientific literature visualization analysis software developed gradually under the background of scientometrics and data visualization. It presents the structure, law and distribution of subject knowledge by means of scientific knowledge map, which can seek out the progress and current research fronts of a certain field. CiteSpace was used to complete clustering, centrality and timeline analysis in this study.

VOSviewer (Version1.6.14) is a software tool for building and visualizing bibliometric networks, these networks can be constructed based on citations, bibliographic coupling, co-citation, or co-authorship relationships. A node represents an element, such as countries, institutions and keywords, etc., and the top 50 of each element were selected to ensure consistency. The greater the width of the links between the nodes, the stronger the collaboration, and the larger the size of the nodes, the more the number of releases.

Pajek is an auxiliary software for adjusting VOSviewer maps, and Scimago Graphica is a chart making software for showing national geographic distribution and literature publication trends.

Data collection and analysis were performed independently by RLL and HD. Only the literature related to the treatment of ASD with AI were included, and the duplicate literature were removed. Any differences were resolved through discussion or by seeking the help of the other authors. Finally, a total of 448 literature were included (Table 1).

## Results

### The quantity and annual trend of published literature

**Figure 1:**
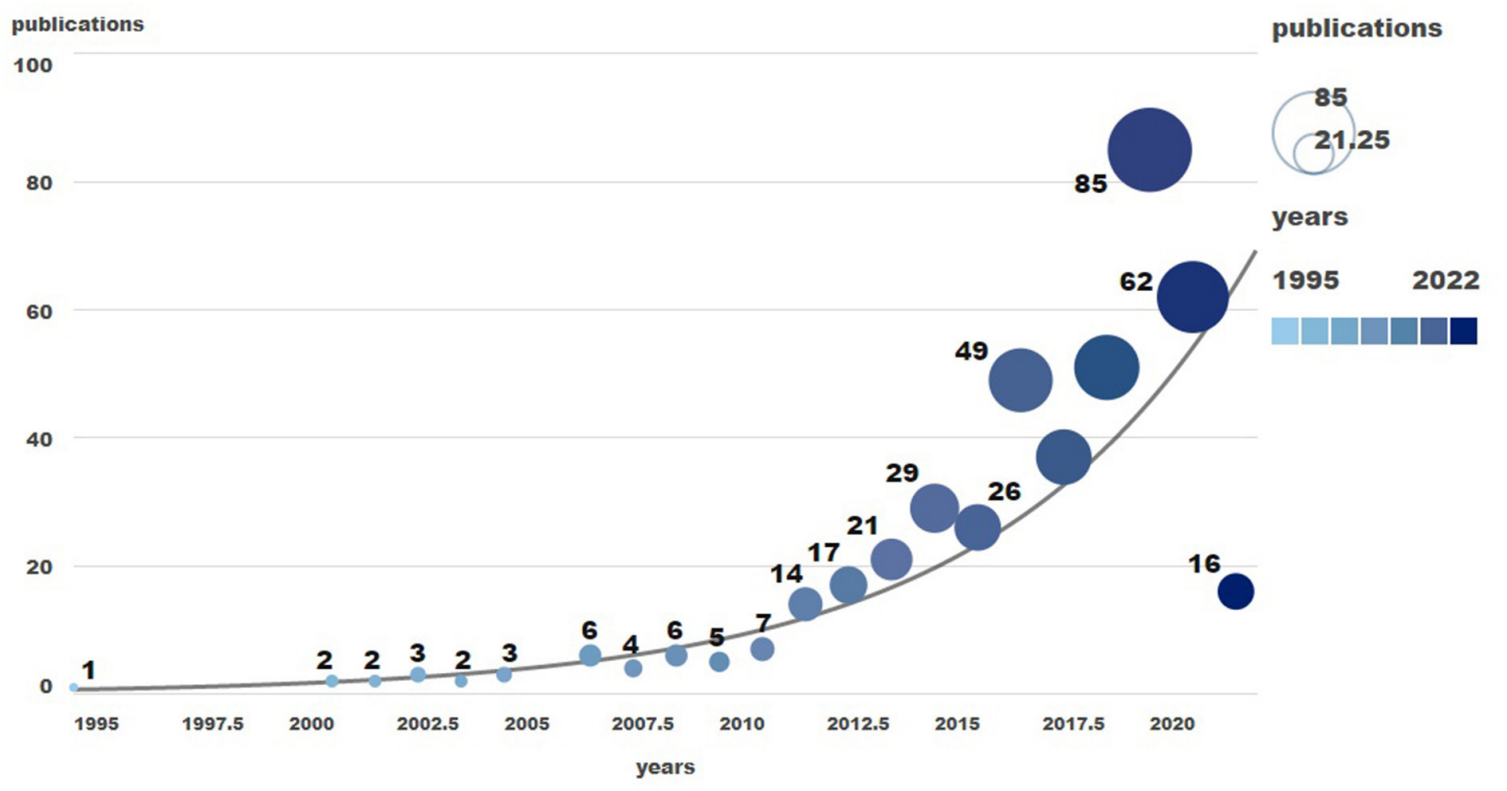
The annual number of publications related to Al on ASD.

A total of 1,298 articles were retrieved from WoS and PubMed, deleting duplicate literature unrelated to AI treatment of ASD. Finally, 448 literature were obtained from 1995 to April 2022. Although there were some fluctuations in the number of literature about AI in the treatment of ASD in WoS and PubMed, the overall number is on the rise (Figure 1). Since 2010, the research results in this field have shown a rapid growth trend, and the number of literature published in 2020 is 85, which is the highest in recent years.

### Ducoments type analysis

A total of 6 types of literature were found (Table 2). Article is the most frequently used literature type, accounting for 45.14% of the total number of publications. Conference paper was the second largest category of literature type (173, 39.05%), the third literature type is review (58; 13.09%).

**Table 2:** Document types related to AI on ASD.

**Ranking**	**Type**	**Counts (%)**	
1	Article	200 (45.14%)	
2	Proceedings paper	173 (39.05%)	
3	Review	58 (13.09%)	
4	Early access	8 (1.80%)	
5	Editorial material	2 (0.45%)	
6	Letter	2 (0.45%)	

### National analysis

Scimago Graphica was used to analyze the geographic cooperation network of countries in this field. A total of 62 countries participated in the study of AI treatment of ASD. It involves Asia, Africa, Europe, North America and South America, among which European countries participate in the highest degree, showing the trend of global cooperation.

**Figure 2:**
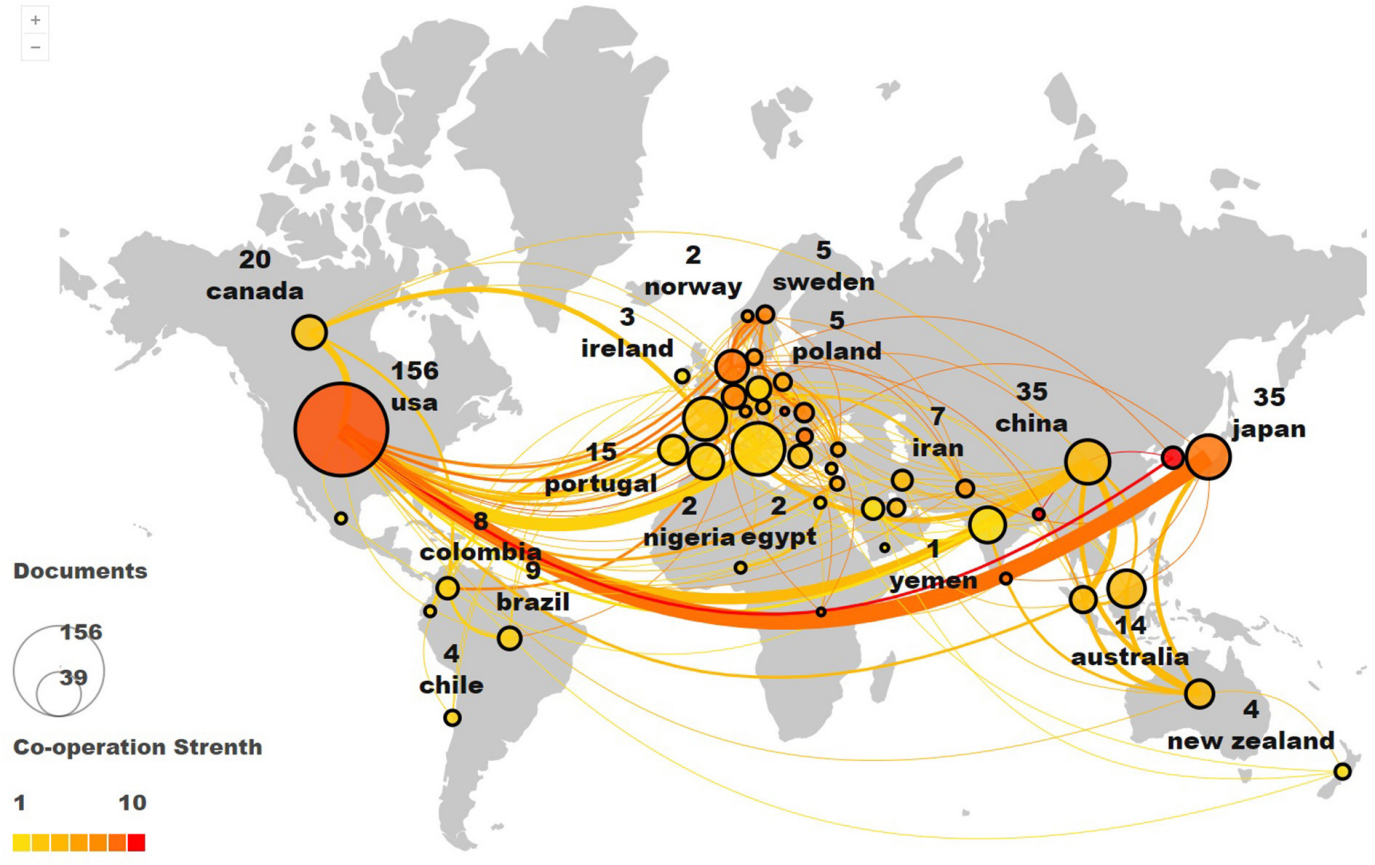
The collaboration network of countries researching Al on ASD.

The United States and Japan have the highest intensity of cooperation. The United States and the United Kingdom, Italy, France, China and the Netherlands also maintain a high cooperation density, which constitutes the largest multi-center cooperation network (Figure 2) in this field. Citespace and Vosviewer are used to analyze the national centrality, the number of publications and the amount of citations. The top three countries of centrality are USA (40), England (34) and France (20). Centrality indicates the degree of attention to the research results. In terms of the volume of articles, USA has the largest number of literature, followed by Italy (50) and England (48). The country with the largest number of citations is USA (3,079), followed by England (1,471) and Italy (579; Table 3). USA has the highest centrality, number of articles and citations, indicating that USA has been active in the field of AI treatment of ASD since 1995, and the research results have attracted wide attention.

**Table 3:** Top 10 publications, centrality and citations of countries related to AI on ASD.

**Rank**	**Publications**	**Countries**	**Centrality**	**Countries**	**Citations**	**Countries**
1	147	USA	40	USA	3097	USA
2	50	Italy	34	England	1471	England
3	48	England	20	France	579	Italy
4	35	China	19	Japan	417	France
5	35	Japan	18	Germany	259	Canada
6	33	France	15	Australia	255	Singapore
7	25	Malaysia	15	Ireland	250	Netherlands
8	23	India	14	Italy	224	Malaysia
9	22	Spain	13	India	215	Japan
10	20	Canada	11	Netherlands	149	China

### Institutional analysis

Citace and vosviewer were used to estimate 310 institutions that have made important contributions to the field. The centrality of Vanderbilt University (9) is the highest, followed by Harvard University (8) and Radboud University Nijmegen (8). Vanderbilt University also has the highest number of posts (20). The top three institutions of cited frequency are Hertfordshire University (630), Vanderbilt University (439) and Paris 06 University (269), and the top three institutions of publication are Vanderbilt University (20), Osaka University (10) and Hertfordshire University (9; Table 4).

**Table 4:** Top 10 publications, centrality, and citations of institutions related to AI on ASD.

**Rank**	**Publications**	**Institutions**	**Centrality**	**Institutions**	**Citations**	**Institutions**
1	20	Vanderbilt Univ	9	Vanderbilt Univ	630	Hertfordshire Univ
2	10	Osaka Univ	8	Harvard Univ	439	Vanderbilt Univ
3	9	Hertfordshire Univ	8	Radboud Univ Nijmegen	269	Paris 06 Univ
4	9	Kanazawa Univ	8	Massachusetts Gen Hosp	180	Eindhoven Univ Technol
5	8	Keio Univ	7	George Washington Univ	168	Univ Technol Mara
6	8	Univ Technol Mara	6	Osaka Univ	131	George Washington Univ
7	8	Harvard Univ	6	Kanazawa Univ	112	Harvard Univ
8	7	Paris 06 Univ	6	Keio Univ	112	Univ Southern Calif
9	7	NatI Inst Aav Ind Sci&Technol	6	Vanderbilt Kennedy Ctr	111	Univ Delaware
10	6	Eindhoven Univ Technol	6	Bambino Gesu Pediat Hosp	109	Radboud Univ Nijmegen

**Figure 3:**
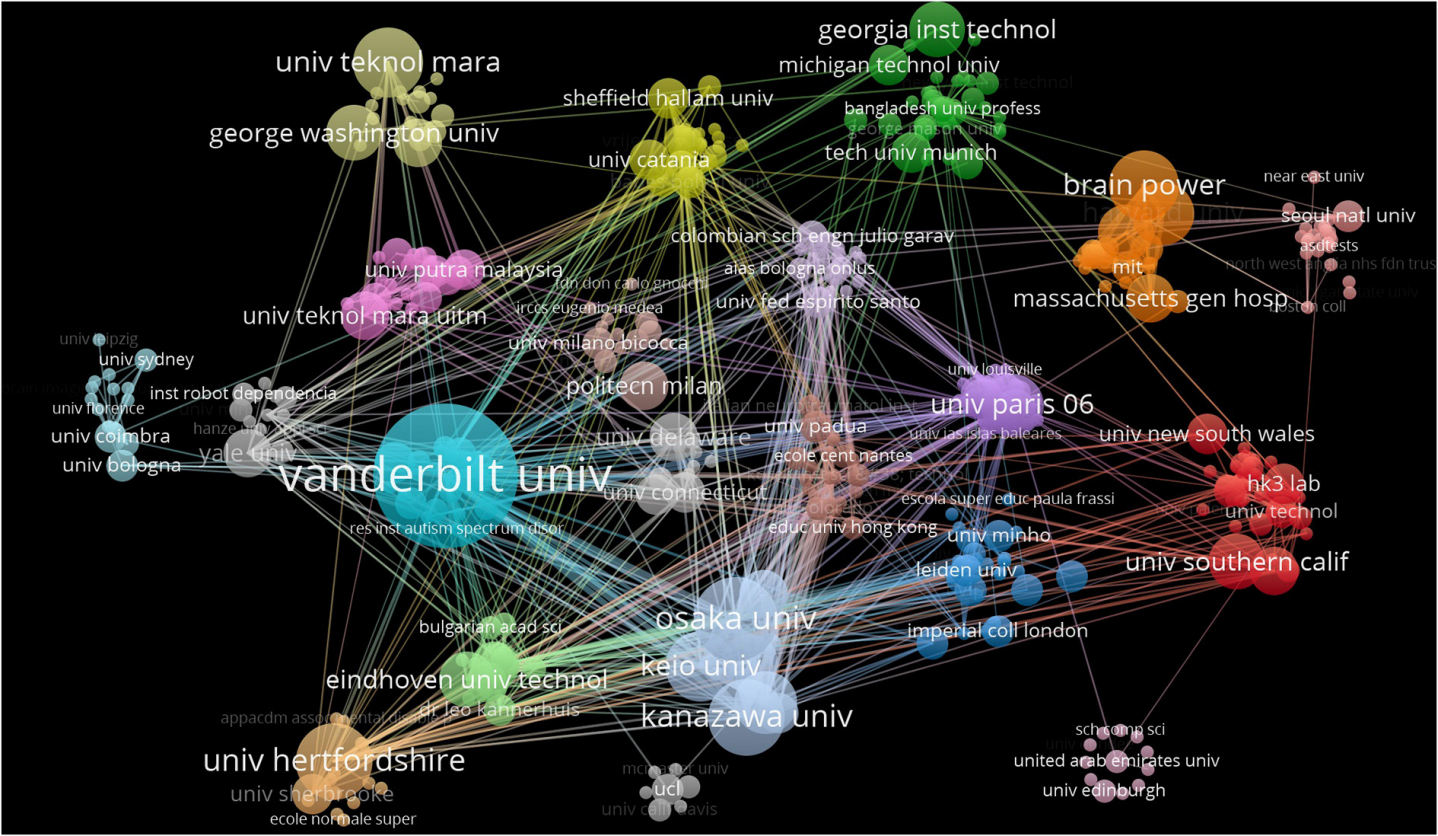
The collaboration network of institutions related to Al on ASD.

The cooperative analysis map shows the main institutional cooperative situation. Taking the top three clusters as an example, blue clustering shows cooperation among Vanderbilt University, Indian Institute of Technology, University of Paris 11 and University of Science & Technology Beijing, white clustering shows cooperation among Osaka University, Kanazawa University, Keio University and Nanyang Technological University, purple clustering shows cooperation among Paris 06 University, Sharif University of Technology, University of Alicante, Islamic Azad University, etc. Vanderbilt University, Hertfordshire University, University of Technology MARA, Osaka University and University of Paris 06 were the key nodes in the collaborative network, promoting a wide range of cooperation among global institutions. Global institutions were characterized by extensive cooperation and small-scale aggregation (Figure 3).

### Author analysis

We analyzed 1,648 authors who have published articles on AI treatment of ASD, 32 of them have published more than five articles, and eight of them have published more than 10 articles. Table 5 lists the top three productive authors, including Warren, Zachary (27), Sakar, Nilanjan (23) and Swanson, Amy (18). The authors with the highest number of citations were Dautenhahn, K, Warren, Zachary, Sakar, Nilanjan. The top three authors in terms of centrality were Sakar, Nilanjan (18), Swanson, Amy (14) and Yoshikawa Yuichiro (13; Table 5).

**Table 5:** Top 10 publications and centrality of authors related to AI on ASD.

**Rank**	**Publications**	**Author**	**Centrality**	**Author**	**Citation**	**Author**
1	27	Warren, Zachary	18	Sarkar, Nilanjan	630	Dautenhahn, K
2	23	Sarkar, Nilanjan	14	Swanson, Amy	447	Warren, Zachary
3	18	Swanson, Amy	13	Yoshikawa Yuichiro	388	Sarkar, Nilanjan
4	16	Kumazaki, Hirokazu	12	Kumazaki, Hirokazu	355	Swanson, Amy
5	14	Zheng, Zhi	12	Kikuchi, Mitsuru	287	Pioggia, Giovanni
6	11	Yussof, Hanafiah	12	Ishiguro, Hiroshi	282	Chetouani, Mohamed
7	10	Yoshikawa Yuichiro	11	Warren, Zachary	282	Cohen, David
8	10	Matsumoto, Yoshio	10	Chetouani, Mohamed	259	Boucenna, Sofiane
9	9	Ishiguro, Hiroshi	10	Cohen, David	242	Tilmont, Elodie
10	9	Kikuchi, Mitsuru	10	Scilingo Enzo Pasquale	228	Zheng, Zhi

**Figure 4:**
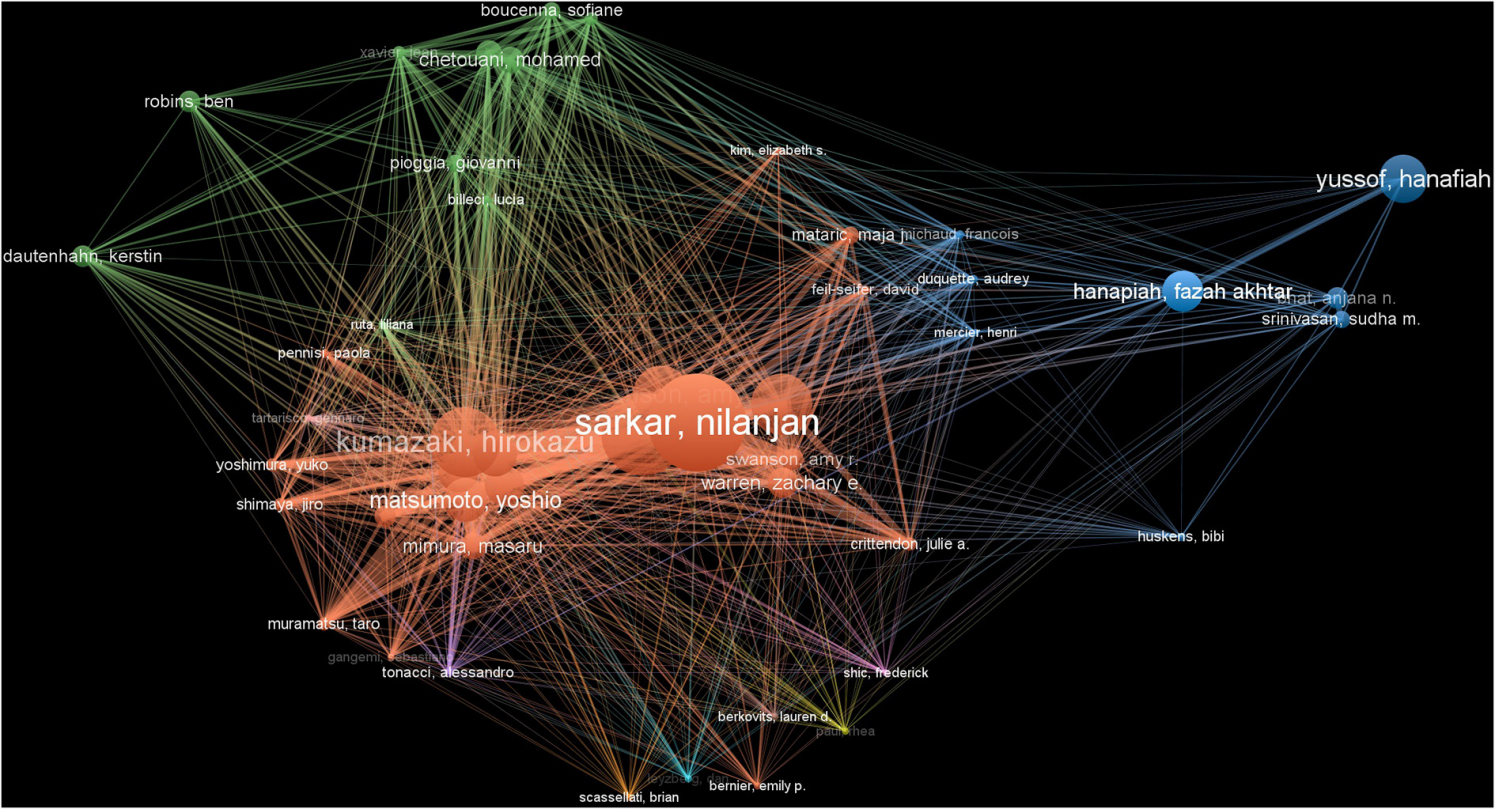
The collaboration network of authors related to Al on ASD.

There was three clusters in the author's cooperative network. The red cluster represents the authors Sakar, Nilanjan, Zheng Zhi and Kumazaki, Hirokazu, whose research focused on the effect of humanoid robots on the ASD patients (13, 14). Boucenna, Sofiane and Chetouani, Mohamed were the major members of the green cluster. Their research focused on the improvement of social behavior and learning ability of patients with ASD (15, 16). The blue cluster mainly included Yussof, Hanafiah and Hanapiah, Fazah Akhtar. They were committed to exploring the application of robots in special education for patients with ASD (17–19) (Figure 4).

### Journal/conference collection analysis

**Figure 5:**
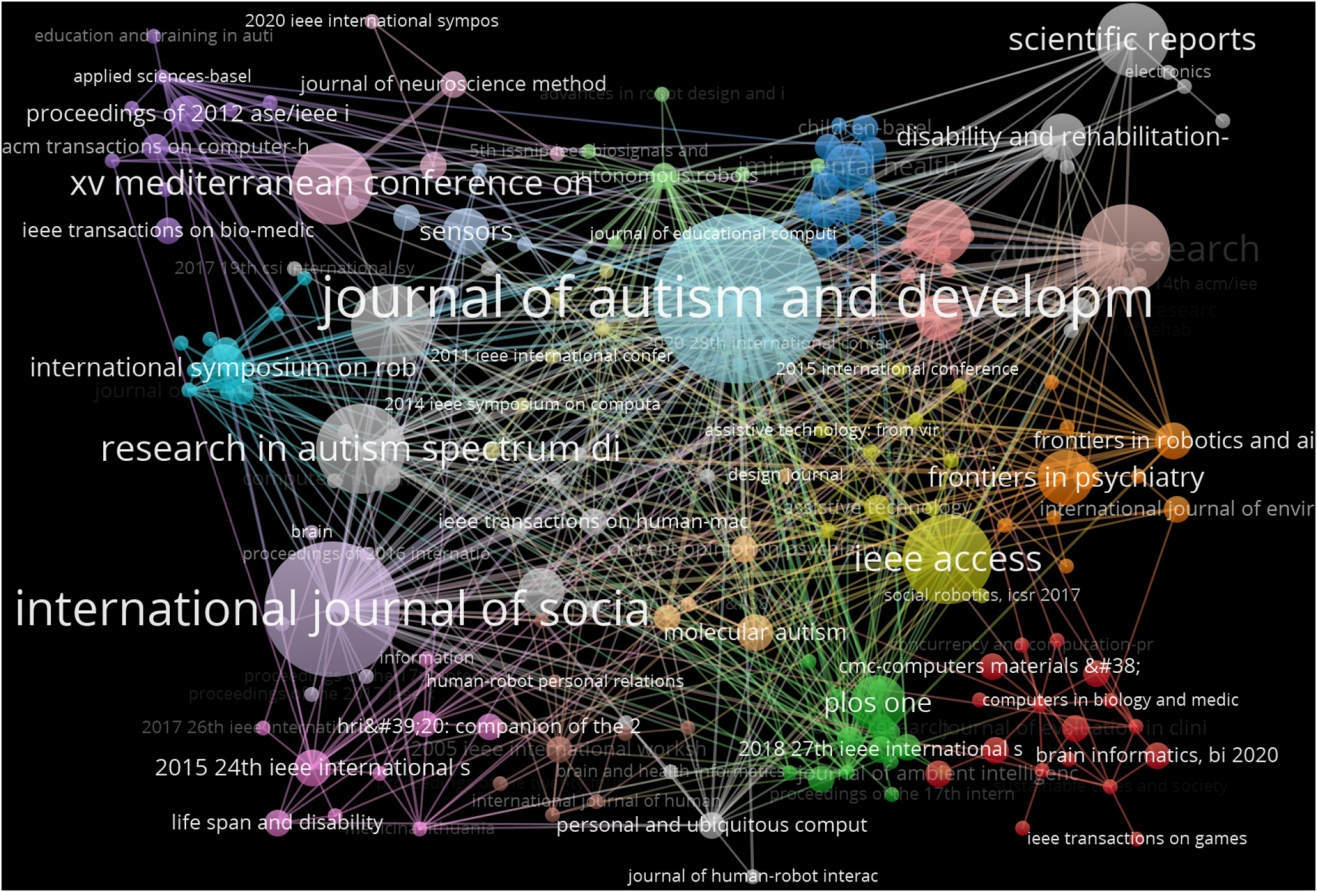
The citation network of journals related to Al on ASD.

VOSviewer and Citespace were used to analyze the top 10 journals as shown in Table 6. Ten of the 271 journals published more than five articles. Journal of Autism and Developmental Disorders published 20 articles, which was the most published journal, followed by International Journal of Social Robotics (15) and Autism Research (9). In terms of cited frequency, Philosophical Transactions of the Royal Society B-Biological Sciences ranked first (451), Journal of Autism and Developmental Disorders ranked second (449), and International Journal of Social Robotics ranked third (230; Table 6, Figure 5).

**Table 6:** Top 10 journals related to AI on ASD.

**Rank**	**Publications**	**Journal**	**Citation**	**Journal**
1	20	J Autism Dev Disord	451	Philos T R Soc B
2	15	Int J Soc Robot	449	J Autism Dev Disord
3	9	Res Autism Spect Dis	230	Int J Soc Robot
4	9	IEEE Access	219	Res Autism Spect Dis
5	7	Scientific Reports	187	IEEE T Neur Sys Reh
6	6	Autism Res	178	Autism Res
7	6	Front Psychol	151	Auton Robot
8	6	IEEE T Neur Sys Reh	138	Cell
9	5	Front Psychiatry	128	Cogn Comput
10	5	Plos One	115	Psychopharmacology

**Figure 6:**
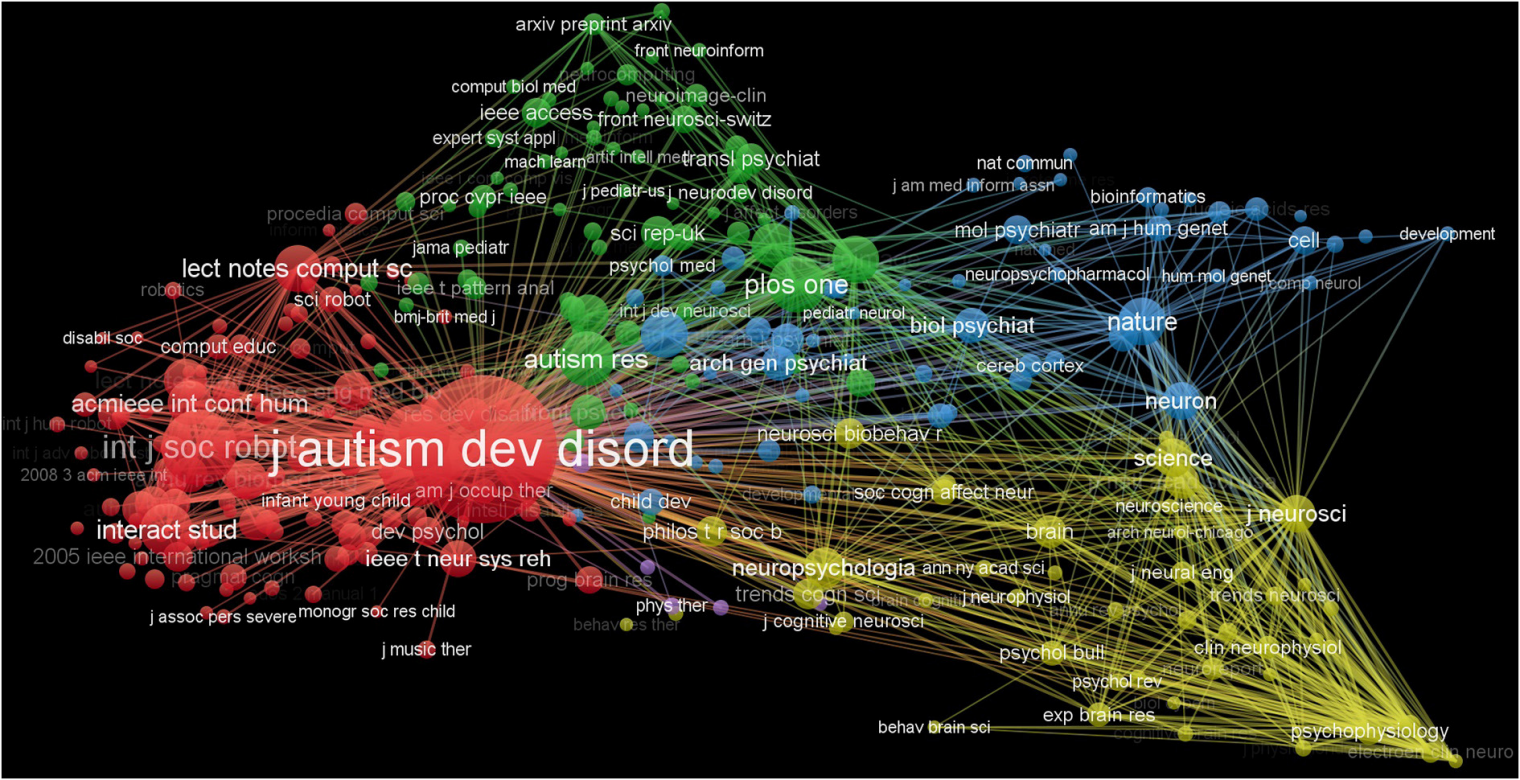
The co-cited network of journals in Al on ASD.

Journal of Autism and Developmental Disorders, Research in Autism Spectrum Disorders and International Journal of Social Robotics are the most frequently co-cited journals. In terms of centrality, American Journal of Psychiatry (69) ranked first, followed by Biological Psychiatry (61) and Brain (52; Figure 6, Table 7).

**Table 7:** Top 10 co-cited journals related to AI on ASD.

**Rank**	**Citation**	**Journal**	**Centrality**	**Journal**
1	293	J Autism Dev Disord	69	AM J Psychiat
2	148	Res Autism Spect Dis	61	Biol Psychiat
3	137	Int J Soc Robot	52	Brain
4	133	Autism	49	Autism
5	116	J Child Psychol Psyc	47	Acmieee Int Conf Hum
6	106	Autism Res	45	J AM ACAD Child Psy
7	100	Plos One	44	Lect Notes Artif Int
8	93	Interact Stud	44	Arch Gen Psychiat
9	90	Annu Rev Biomed Eng	43	J Child Psychol Psyc
10	72	Diagn Stat Man Ment	43	Plos One

**Figure 7:**
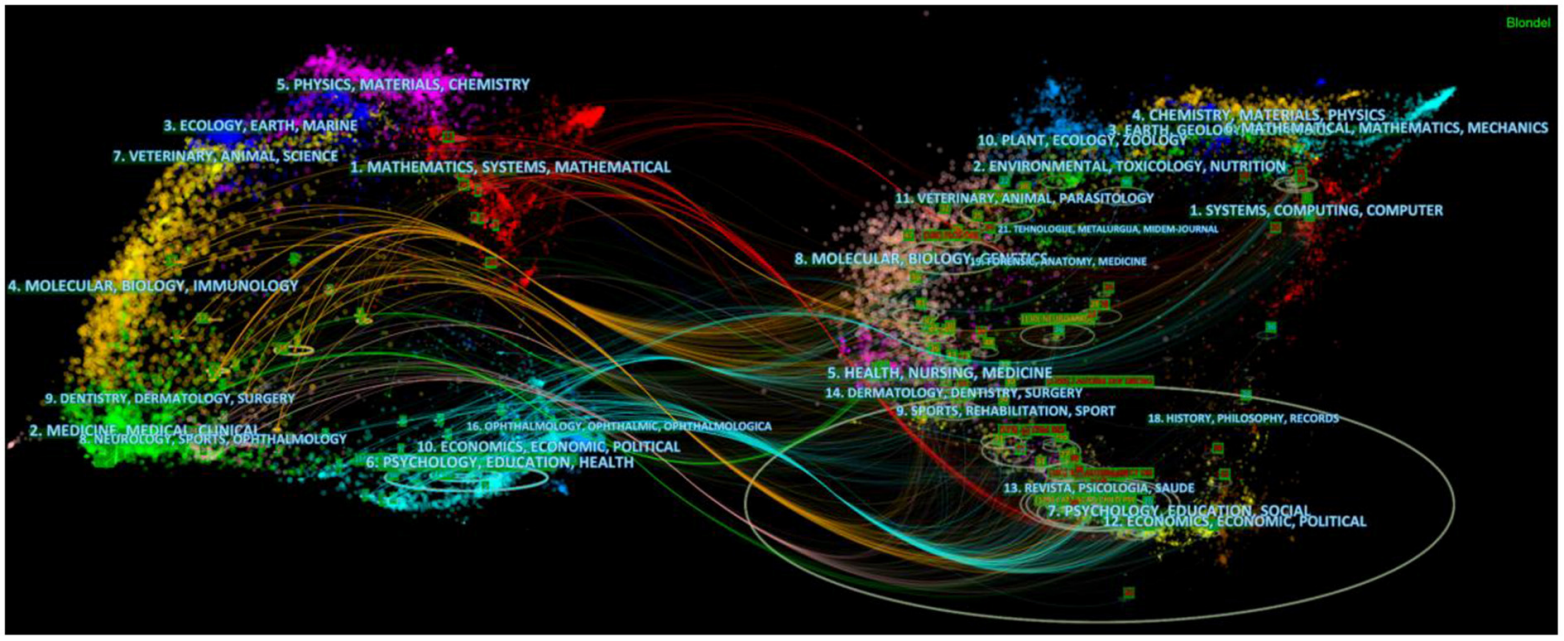
The dual-map overlays of journals in Al on ASD.

Figure 7 illustrated the citation relationship between the cited journals and the citing journals. we found that there was a reciprocal citation relationship between the literature involved in psychological, educational, social fields and those in molecular biology, genetic, computer fields, which illustrated that researchers have tried to find out the relationship between specific genes and psychological/social disorders of ASD patients from the perspective of molecular biology (20, 21) and explore the psychological and educational issues of ASD patients with the help of computer technology (22).

Conference proceedings remains an important way to understand the progress of research in this field. the american society of electronic, electrical and automotive engineering (ieee) is an major sponsor of international conferences. from 2002 to 2021, a total of 69 international conferences have been held to discuss the intelligent diagnosis of ASD (23), improvement of social skills of patients with ASD (24) and the application of brain-computer interface (BCI) (25), etc. In 2012, International Symposium on Robots and Intelligent sensors held in Malaysia (IRIS) to explored whether robots can improve the stereotyped behaviors of children with ASD (26), which received a higher citation frequency (167).

### Reference analysis

**Figure 8:**
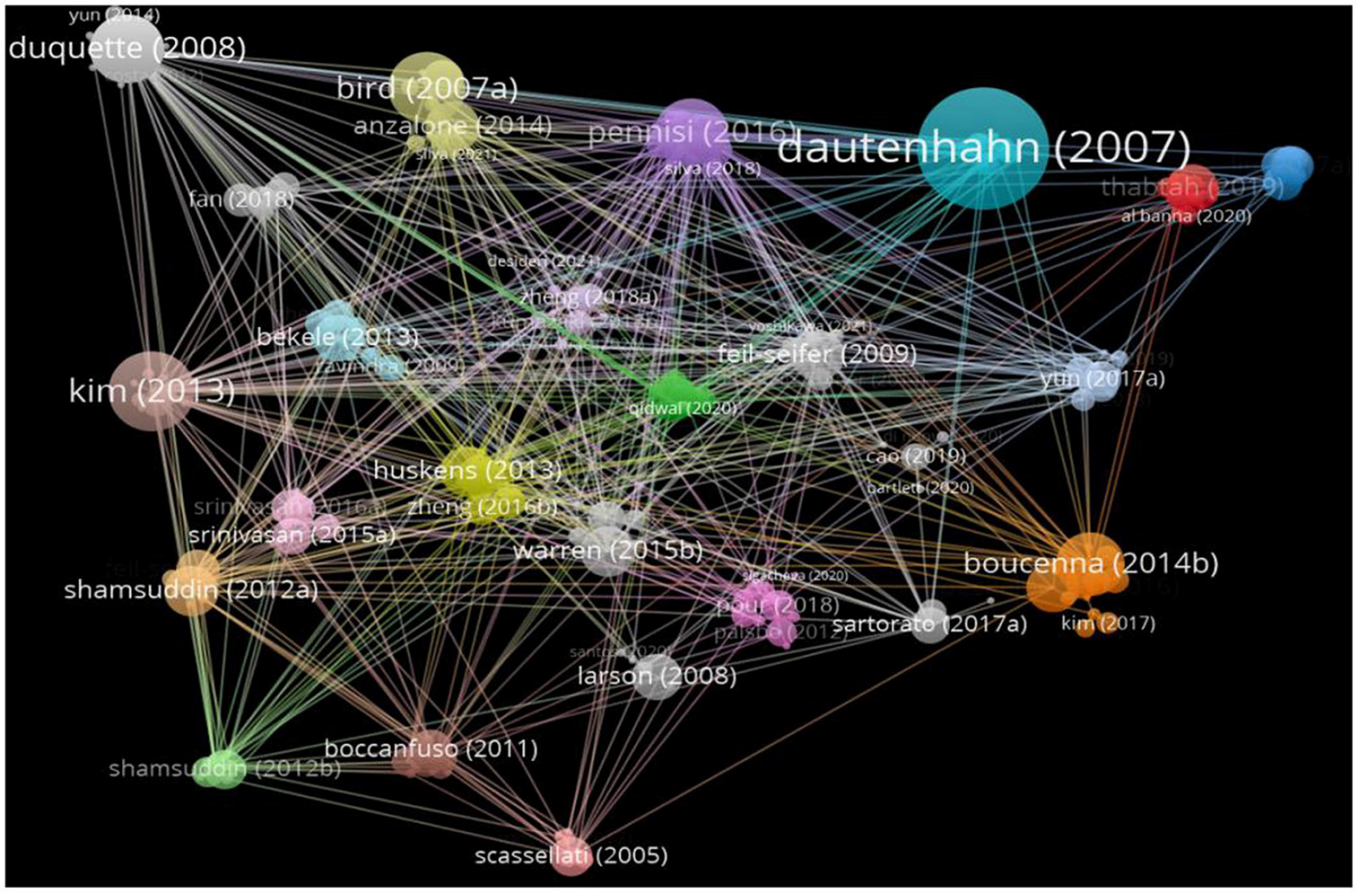
The network of co-cited references related to Al on ASD.

CiteSpace was used to analyze the citation count and centrality. The top 10 cited references are shown in Table 8 and Figure 8. A total of 448 articles were included, of which 143 were cited more than 10 times. Dautenhahn (22) had the highest citation counts (451), followed by Bird et al. (27) and Bird et al. (28). Their studies focused on imitative and social impairments in ASD patients, suggesting that social robots may be a useful tool for promoting social skills in ASD patients (27, 28). Seven of the top 10 most cited articles discussed the impact of AI robots on the social interaction ability of ASD patients (Figure 8, Table 8).

**Table 8:** Top 10 cited references related to AI on ASD in terms of centrality.

**Rank**	**Citations**	**Cited reference**	**Representative author (publication year)**
1	451	Socially intelligent robots: Dimensions of human–robot interaction	Dautenhahn (2007)
2	192	Social Robots as Embedded Reinforcers of Social Behavior in Children with Autism	Kim (2013)
3	156	Intact automatic imitation of human and robot actions in autism spectrum disorders	Bird G (2007)
4	140	Autism and Social Robotics: A Systematic Review	Pennisi (2016)
5	138	Histone Acetylome-wide Association Study of Autism Spectrum Disorder	Wen Jie Sun (2016)
6	133	Exploring the use of a mobile robot as an imitation agent with children with low-functioning autism	Duquette (2008)
7	123	Interactive Technologies for Autistic Children: A Review	Boucenna S (2014)
8	115	Biomarkers in autism spectrum disorder: The old and the new	Ruggeri (2014)
9	84	Real-Time Mental Arithmetic Task Recognition From EEG Signals	Qiang Wang (2013)
10	79	How children with autism spectrum disorder behave and explore the 4-dimensional (spatial 3D+time) environment during a joint attention induction task with a robot	Anzalone (2014)

### Keyword analysis

**Figure 9:**
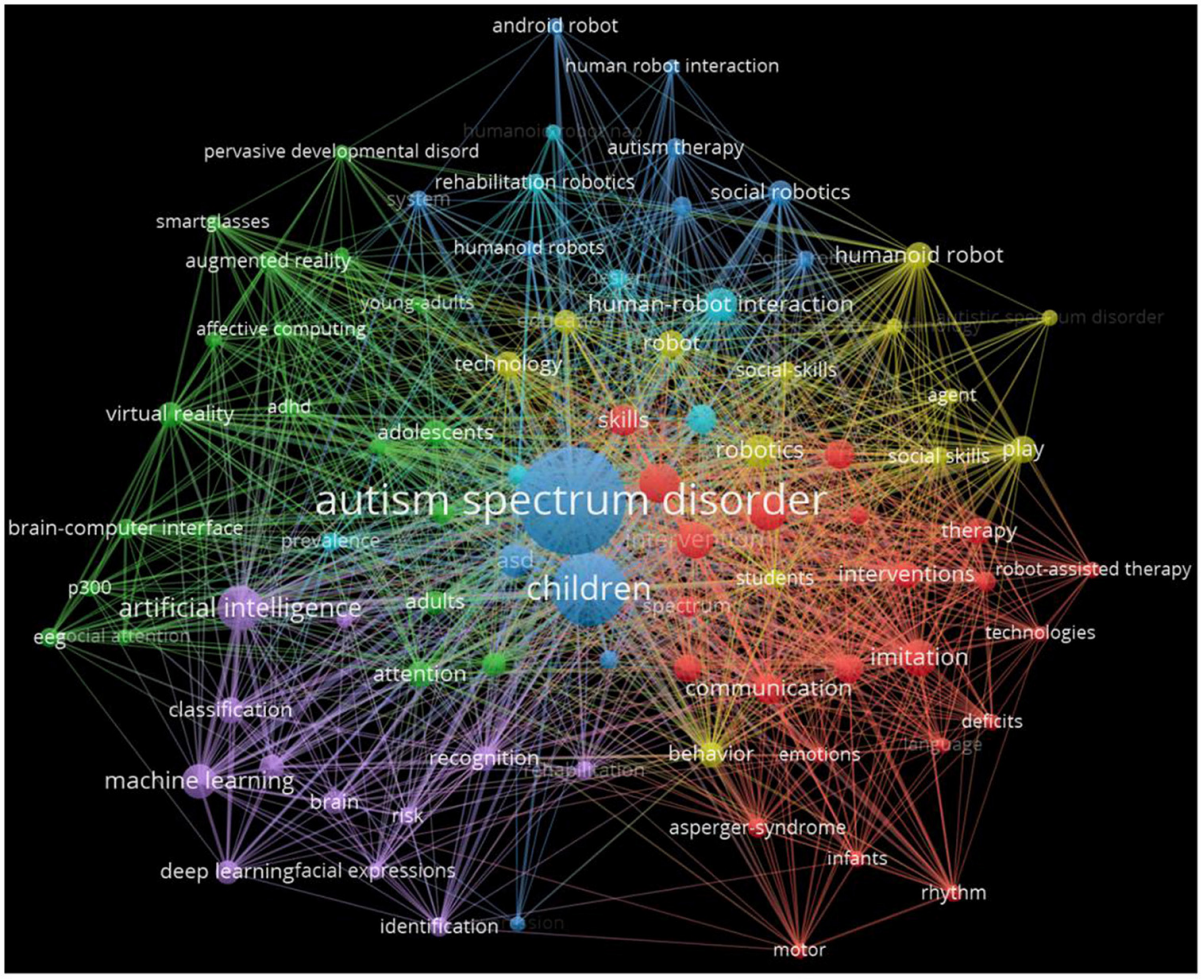
The network of co-occurence keywords related to Al on ASD.

This study selected the top 10 keywords with the strongest co-occurrence frequency, which is helpful to understand the research hotspots in this field from 1995 to 2022. “Autism spectrum disorder” is the keyword with the highest frequency of co-occurrence (301), followed by “Children” and “Artificial Intelligence”. In terms of centrality, “Children” has the highest centrality (69), followed by “Brain Computer Interface” and “Attention Deficit” (Figure 9; Table 9).

**Table 9:** Top 10 keywords related to AI on ASD.

**Rank**	**Co-occurence**	**Keyword**	**Centrality**	**Keyword**
1	301	Autism spectrum disorder	69	Children
2	140	Children	57	Brain computer interface
3	51	Artificial intelligence	46	Attention deficit
4	41	Individuals	43	Autism spectrum disorder
5	37	Joint attention	43	Intervntion
6	36	Intervention	38	Imitation
7	34	Imitation	38	Adolescent
8	32	Machine learning	35	Biopoar disorder
9	30	Robotics	34	Humanoid robot
10	29	Human-robot interaction	32	Socia robot

Citespace is used to complete the cluster analysis, the cluster map classifies the keywords according to the correlation, and the complex keywords are divided into several characteristic clusters. The data in the upper left corner shows two indexes: Modularity and Mean Silhouette. When the Modularity is more than 0.3, it shows that the clustering structure is significant, and when the Mean Silhouette reaches 0.7, the clustering result is convincing. In this study, the Modularity is 0.657 and the Mean Silhouette is 0.871, so the clustering structure of the cluster map is very significant, and the result is convincing.

**Figure 10:**
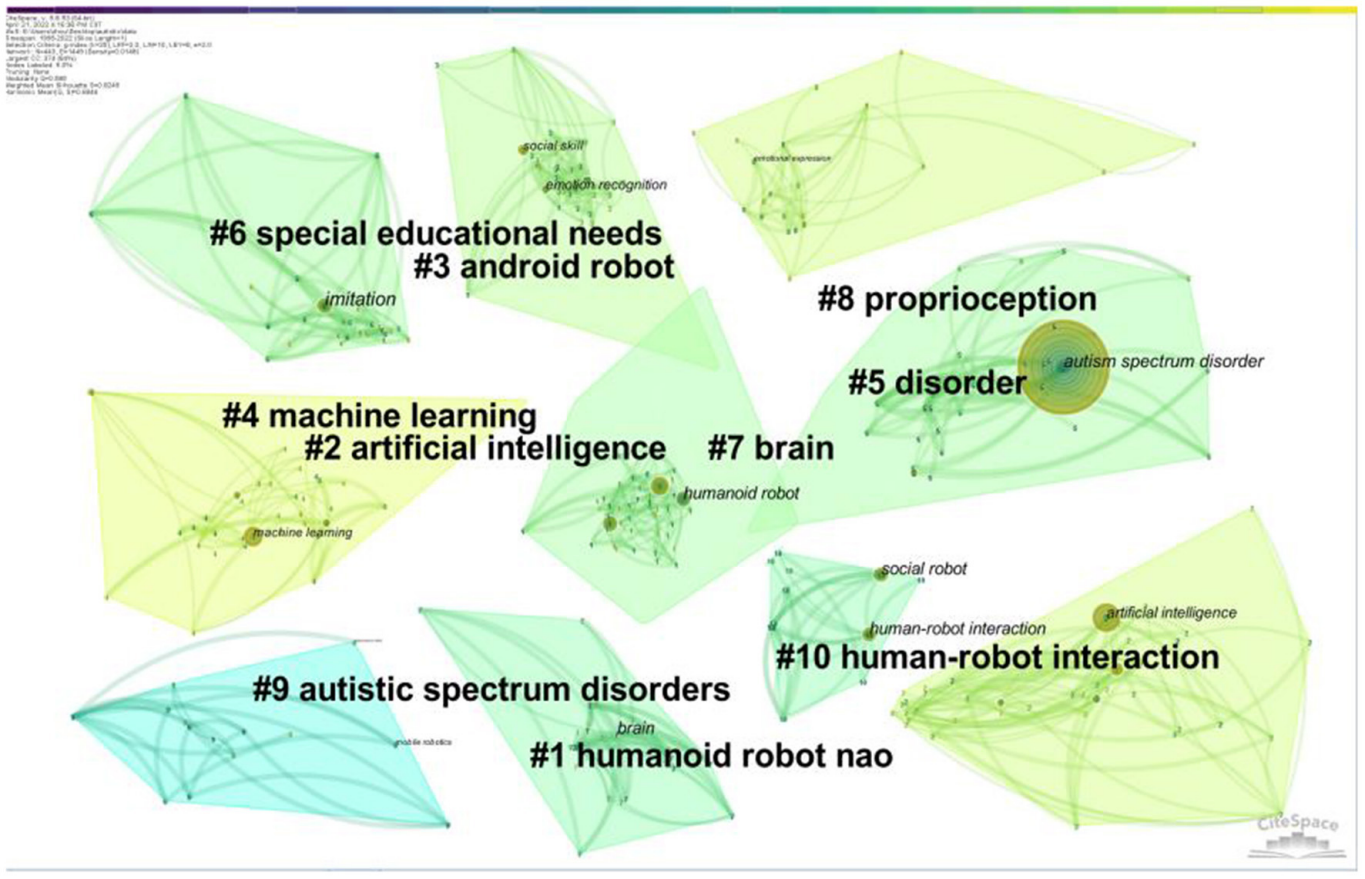
The cluster map of co-occurence keywords related to Al on ASD.

“Special educational needs” “proprioception” “human-robot interaction” “machine learning” and “humanoid robot” were the main clusters. “Disorder” was the largest cluster, with 54 members, and Autism and social robotics: A systematic review (29) was the most frequently cited article under this cluster. This article makes a systematic review of robot application in ASD research in the decade from 2006 to 2016 (Figure 10).

**Figure 11:**
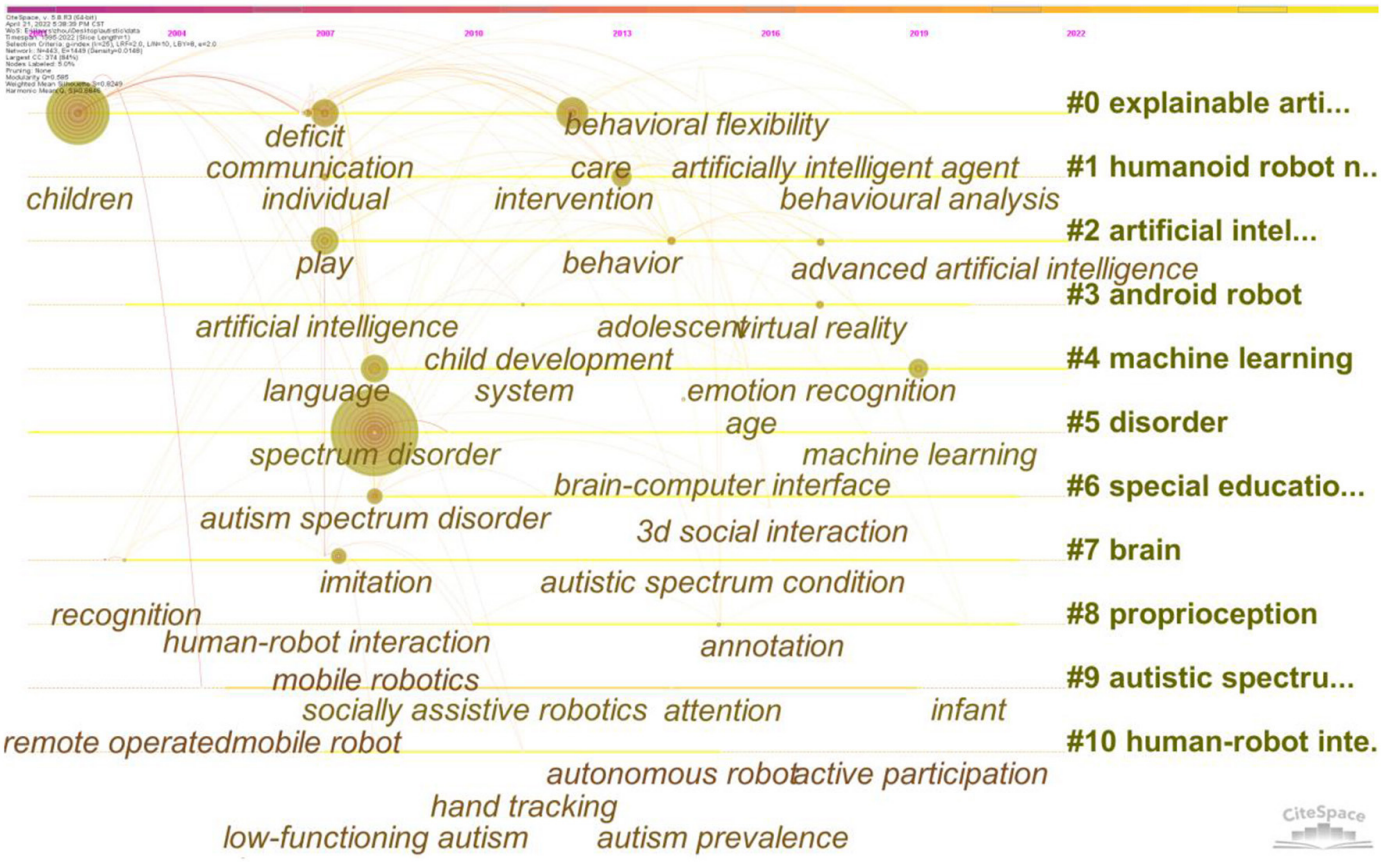
The cluster timeline view network of keywords related to Al on ASD.

Figure 11 presents a key words cluster time line diagram analyzing the time in which the top key words appear and continue to be of interest, helping to understand the research hotspots in the field at different time periods. “children” after their first appearance in 2002, and “deficit”, “play”, “communication” maintained a high degree of association, indicating that the absence of social functioning in children with ASD is an urgent problem (30, 31). After 2008, “Artificial intelligence” began to receive much attention, and after 2012, keywords such as “behavior disability”, “eomotion recognition” emerged, indicating that ASD patients' behavioral activity and emotion recognition has become the focus of attention (32, 33).

**Figure 12:**
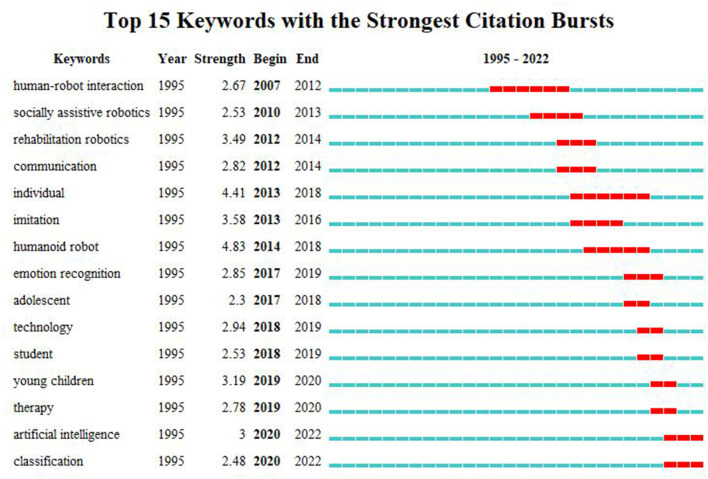
The top 15 keywords with the strongest citation bursts related to Al on ASD.

Burst keywords were analyzed using CiteSpace. Burst Keywords means this key word has received highly attention in a certain time period. “human robot interaction” was the key word of the earliest burst, maintaining a high intensity outbreak in 2007–2012. Since 2007–2018, the key words “socially assistive robotics” “rehabilitation robotics” “humanoid robot” maintained a high outbreak value (34, 35). “emotional recognition” “young children” “student” and “classification” have been the most focused keywords in the last 5 years (Figure 12).

## Discussion

A bibliometric analysis was used to analyze the studies on AI treatment of ASD from 1995 to April 2022. Since the publication of the first paper in 1995, the number of papers in this field continues to grow, with a slight decreasinng trend after 2020 to the present, which may be the outbreak of COVID-19 leading to the reduction in global cooperation, however, according to current trend analysis, Research on AI treatment of ASD will continue to show an overall increasing trend in the coming years.

In the field of AI for the treatment of ASD, global countries show a trend of extensive participation, especially European countries have the highest level of participation and show obvious clustering, one of the possible reasons is that European visa policy makes it easy for European research institutions to recruit researchers from other European countries, another reason is that European Research Council provides a large number of opportunities for researchers from different European countries to cooperate (36).

The United States and Japan are central members of a worldwide collaborative network, focusing in the last 10 years on studying the imitation capability of robots (37, 38), exploring their use in ASD patients (39, 40), and providing an exploration of the “humanness” of robots (14). The top 10 authors are cited only 20% of the total number of citations. This means that a large number of researchers are still involved in the research of artificial intelligence treatment ASD. We found that authors with higher publications do not necessarily have higher centrality and citation frequency, indicating that authors' academic influence is affected by many factors. From 1995 to 2022, the top 10 journals involved the field of robotics and mental cognition, which shows that the role of robots in improving the psychological and cognitive impairment of ASD patients has received extensive attention (41–43). In the past 26 years, “Children” was the keyword with high co-occurrence frequency, indicating that children are the key population of ASD research (44–46). In recent years, the incidence of ASD in children has been on the rise, which may be related to the improvement of parents' awareness of the disease and the progress of medical diagnosis, early screening and intervention are beneficial to the rehabilitation of ASD (47, 48). “Humanoid Robot” is the keyword with the highest burst intensity, and more and more studies have shown that intelligent robots can help improve the emotional cognition ability of ASD patients (49), social interaction ability (50), joint attention (13). More interestingly, ASD patients seem to have a higher affinity and acceptance of intelligent robots than human therapists (37, 51). Therefore, in recent years intelligent robots have been widely used in the special education of ASD patients to help them gradually integrating into the society. In addition, the “Brain-Computer Interface” (BCI) is an AI technology that has received much attention recently. It relies on machine learning algorithms to decode brain signals (52) and is considered a potential ASD rehabilitation method (53–55).

## Limitations

As the Citespace software can not directly identify the literature of the PubMed database, we need to convert the PubMed data into WoS format, which may lead to the recognition bias of some of the data; In addition, the text content of some pictures is incomplete due to the limitations of the software, but it does not affect reading, the software needs to be further improved in the future.

## Conclusion

In conclusion, this study revealed the research status of AI in the treatment of ASD, as well as the hot spots and research fronts. The use of AI for the treatment of ASD is a relatively new field, but there is no doubt that this field will continue in rapid updates driven by the dual need for global markets and technological advances. AI will be applied to ASD in more diverse and user-friendly forms, such as wearable devices and brain machine interface technology, which has become a new wave of research direction and expected to be a potential method to cure ASD in the future. The restoration of social function of ASD patients will continue to be the focus of global research.

## Data availability statement

The original contributions presented in the study are included in the article/supplementary material, further inquiries can be directed to the corresponding authors.

## Author contributions

XT and XZ contributed to the concept and design of the research and wrote part of the manuscript. Data collection and analysis was conducted by RL and HD. SZ and SW wrote the first draft of the manuscript. All the authors contributed to the revision of the manuscript, reading, and approving the submitted version.

## Funding

This study was supported by Yunnan Provincial Department of Science and Technology Biomedical Key Project (Grant No. 202102AA100016) and Yunnan Basic Research Traditional Chinese Medicine Joint Special Project (Grant Nos. 202001AZ070001-002 and 202101AZ070001-059).

## Conflict of interest

The authors declare that the research was conducted in the absence of any commercial or financial relationships that could be construed as a potential conflict of interest.

## Publisher's note

All claims expressed in this article are solely those of the authors and do not necessarily represent those of their affiliated organizations, or those of the publisher, the editors and the reviewers. Any product that may be evaluated in this article, or claim that may be made by its manufacturer, is not guaranteed or endorsed by the publisher.
